# The Optimization of a Novel Hydrogel—Egg White-Alginate for 2.5D Tissue Engineering of Salivary Spheroid-Like Structure

**DOI:** 10.3390/molecules25235751

**Published:** 2020-12-06

**Authors:** Yuli Zhang, Hieu M. Pham, Jose G. Munguia-Lopez, Joseph M. Kinsella, Simon D. Tran

**Affiliations:** 1 Faculty of Dentistry, McGill University, 3640 University Street, Montreal, QC H3A 0C7, Canada; yuli.zhang@mail.mcgill.ca (Y.Z.); mikehieupham@hotmail.com (H.M.P.); jose.munguia-lopez@mail.mcgill.ca (J.G.M.-L.); 2 Department of Bioengineering, McGill University, 3480 University Street, Montreal, QC H3A 0E9, Canada; joseph.kinsella@mcgill.ca

**Keywords:** tissue engineering, hydrogel, alginate, egg white, salivary gland

## Abstract

Hydrogels have been used for a variety of biomedical applications; in tissue engineering, they are commonly used as scaffolds to cultivate cells in a three-dimensional (3D) environment allowing the formation of organoids or cellular spheroids. Egg white-alginate (EWA) is a novel hydrogel which combines the advantages of both egg white and alginate; the egg white material provides extracellular matrix (ECM)-like proteins that can mimic the ECM microenvironment, while alginate can be tuned mechanically through its ionic crosslinking property to modify the scaffold’s porosity, strength, and stiffness. In this study, a frozen calcium chloride (CaCl_2_) disk technique to homogenously crosslink alginate and egg white hydrogel is presented for 2.5D culture of human salivary cells. Different EWA formulations were prepared and biologically evaluated as a spheroid-like structure platform. Although all five EWA hydrogels showed biocompatibility, the EWA with 1.5% alginate presented the highest cell viability, while EWA with 3% alginate promoted the formation of larger size salivary spheroid-like structures. Our EWA hydrogel has the potential to be an alternative 3D culture scaffold that can be used for studies on drug-screening, cell migration, or as an in vitro disease model. In addition, EWA can be used as a potential source for cell transplantation (i.e., using this platform as an ex vivo environment for cell expansion). The low cost of producing EWA is an added advantage.

## 1. Introduction

Patients treated with radiotherapy for head/neck cancer (≈500,000 new patients annually worldwide) and patients with Sjögren’s syndrome (the 2nd most prevalent autoimmune disease) experience decreased saliva flow due to the loss of the salivary gland (SG) function [1,2,3,4]. SG hypo- or dysfunction results in speech and swallowing difficulties, dry mouth, altered taste perception, dental caries, oropharyngeal infections, and mucositis. Current treatments are only palliative and consist of sipping water constantly, using oral moisturizing products or pharmaceutical agents to stimulate the remaining acinar cells to produce saliva [5]. The use of three-dimensional (3D) culture that simulates the native cell/organoid microenvironment is a reliable strategy to study ex vivo cellular biology aiming at treatments for diseases [6,7]. A critical challenge in 3D organoid culture is developing an appropriate scaffold for specific cells where the microenvironment allows the cells to reorganize into a functional organoid inducing cells to proliferate, migrate, and differentiate [8].

Hydrogels have been used for a variety of biomedical applications, including being among the most common scaffolds for tissue engineering. Hydrogels are polymers capable of holding high quantities of water (more than 75% *w*/*v*) inside of their 3D structure; they are widely used to create 3D scaffolds as a synthetic surrogate for the native cell’s ECM [9,10]. Hydrogels provide ideal cellular microenvironments for cell proliferation and differentiation due to their highly hydrated environments, resemblance to natural anatomy, and crosslinked networks providing porosity [8,11]. Natural polymers, derived from sources such as algae, animals, and micro-organisms, have frequently been used to make hydrogel scaffolds for tissue-engineering applications owing to their biocompatibility, inherent biodegradability, and anchoring sites allowing cells to attach to their surfaces to drive cell division, adhesion, and migration in a 3D environment [12,13]. When compared with natural polymers, synthetic polymers derived either from lactic acid, caprolactone, or glycoside monomers, possess more reproducible chemical, mechanical, and physical properties, which are critical for the fabrication of tissue-engineering scaffolds [10,14]; however, a setback for synthetic polymers is that they lack adhesion sites necessary for cell adhesion, migration, and differentiation [13]. Recently, bioactive synthetic hydrogels have emerged as promising hydrogel scaffolds because they can be molecularly tailored with block structures, molecular weights, mechanical strength, and biodegradability, in addition to their ability to mimic the natural ECM to provide a desirable cellular environment for supporting cell growth.

Based on the chemical properties of hydrogels, they can be classified as follows: (a) homopolymeric hydrogels, comprised of a single species of monomer, which is a basic structural unit employed to build the polymer network [15]. Homopolymers may have a crosslinked skeletal structure depending on the nature of the monomer and polymerization technique. (b) Copolymeric hydrogels, comprised of two or more different monomer species with at least one hydrophilic component, arranged in a random, block, or alternating configuration along the chain of the polymer network [16]. (c) Multipolymer interpenetrating network (IPN) hydrogel, is made of two independents crosslinked synthetic and/or natural polymer components, contained in a network form. In a semi-IPN hydrogel, one component is a crosslinked polymer, while another component is a non-crosslinked polymer that remains trapped within the 3D structure [17,18].

Except for supporting cell immobilization and growth factor delivery, hydrogels have great potential to be “smart” scaffolds, which can be remotely controlled by external stimuli such as temperature, ionic strength, or enzymatic environment [19,20,21]. Among all the hydrogels, Matrigel is considered the gold standard for 3D scaffold systems due to its commercial availability and physicochemical and biological properties that can mimic the ECM. Matrigel is a protein-based ECM extracted from Englebreth-Holm-Swarm tumors in mice, comprised of laminin, collagen IV, and enactin, which costs around $16/mL [22]. Considering that traditional 3D cell culture requires at least 10 mL/sample, the total expenses increase exponentially. For this reason, it is enticing to seek a less expensive yet still biocompatible alternative to be used as 3D culture scaffolds.

Among the widely available types of IPN hydrogels, alginate is one of the most common polymers used as a 3D matrix due to its biocompatibility, relatively low production cost, and mild gelation through the addition of divalent cations such as Ca^2+^ [23]. Alginate is an inert carbohydrate (it cannot interact with biological entities provoking alteration in their biological functions) copolymer comprised of 1–4 linked β-d-mannuronic acid (M) and α-L-guluronic acid (G) isolated from brown seaweed and some bacterial species [24]. Divalent ions (i.e., Ca^2+^, Ba^2+^, Sr^2+^) are used as a crosslinker to form an “egg-box” structure by the interaction with blocks of G monomers, producing a stiff and stable 3D structure [25,26], thus allowing mechanical modification of the alginate’s properties to mimic the ECM stiffness of particular tissues [24,27]. Alginate is purified by a multi-step extraction procedure yielding high-quality materials that are inert in mammals [28]. It has been shown that no significant inflammatory response is observed when gels formed from commercially available, highly purified alginate are subcutaneously injected into mice [29].

Since alginate lacks bioactive sites for cell adhesion, proteins or peptides containing adhesion sequences are commonly used in alginate biomaterials; thus, egg white (EW) has been noted as a promising material to be used in conjunction with alginate [13]. In recent years, many studies showed that the protein-dense component of the EW is a viable scaffold due to its great advantages in cellular attachment, differentiation, and proliferation [30,31]. EW is comprised of an albumen layer, which is mainly composed of ovalbumin (<50%) and other structural proteins that resemble ECM proteins (such as collagen), acting as a substrate for cellular attachment [6,32]. All these EW proteins make EW a great candidate to be used as complementary material in alginate hydrogels allowing the creation of 3D microenvironments that mimic native mammalian ECM.

EW-based biomaterials have been proven advantageous in tissue engineering: (i) it can provide sufficient nutrition for chicken gametes to grow into a baby chick; (ii) its transparency makes it convenient to monitor and record the changes in cell morphology; and (iii) it is inexpensive and widely available [32,33,34,35]. A study reported that epithelial breast tumor cell lines grown on EW have comparable phenotypes as those grown on Matrigel [32]. Similar observations were found in a study comparing human umbilical vein endothelial cells grown on EW and Matrigel [6]. These studies highlight the feasibility of using egg white as an alternative biomaterial to Matrigel.

One challenge for EWA hydrogels is the instantaneous formation of gels when crosslinked via divalent ion solutions at room temperature, which produces gels that have rough surfaces and are also highly heterogeneous, in addition to their properties being difficult to control [36]. The use of frozen alginate or calcium/solvent solution has been proposed as a methodology to create more homogeneous crosslinked hydrogels. EWA’s viscosity increases with decreasing temperature; thus, a more homogeneous hydrogel can be maintained with the 3D structure that was originally intended [37,38].

**Figure 1:**
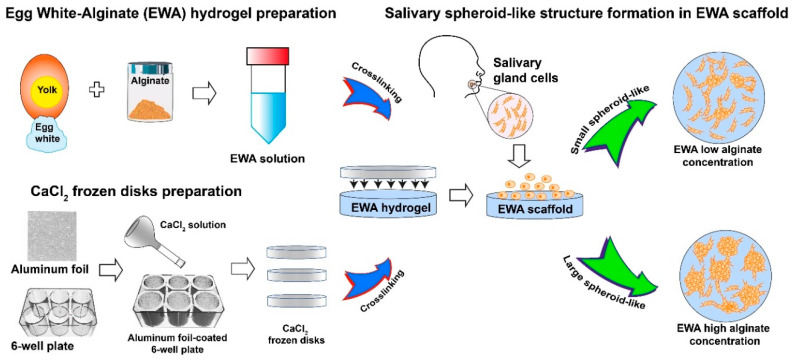
Schematic illustration showing the EWA hydrogel preparation, frozen CaCl_2_ disk, and salivary gland spheroid-like structure formation.

In this study, we present the use of frozen CaCl_2_ disks that allows alginate and EW to crosslink in a more controlled manner through the slow diffusion of Ca^2+^ ions (Figure 1). This technique improves the smoothness of the EWA hydrogel surfaces as well as significantly reduces the formation of macro pits and bubbles generated by the addition of the CaCl_2_ solution using a slow-dispensing micro-pipetting technique. Furthermore, the use of frozen CaCl_2_ disks allows for better cell distribution across the EWA scaffold surface due to the steady homogenous crosslink. Increasing the alginate concentration in the EWA scaffold, SG spheroid-like structure formation is promoted with high cell viability. We used the term “spheroid-like structure” to refer to cell aggregates in a 3D structure. Besides, and to be more accurate, we use the term “2.5D culture” instead of “3D culture”, which has been reported and defined as a culture method that cells are grown on 2D surfaces but partially exhibited similar characteristics to the cells grown in 3D, for example, they grow as cell aggregates [39].

Our proposed EWA hydrogel has the potential to be an alternative 2.5D/3D-culture scaffold that can be used for studies on drug-screening, cell migration, or as an in vitro disease model. In addition, EWA can be used as a potential source for cell transplantation (i.e., using this platform as an ex vivo environment for cell expansion). The low cost for producing EWA is an added advantage.

## 2. Results and Discussion

### 2.1. Scaffold Development

Figure 1 shows the general protocol followed to create EWA scaffolds using frozen CaCl_2_ disks as the crosslinker, and the formation of salivary gland spheroid-like structures.

Egg white material is extracted from eggs and mix with different concentrations of alginate to create various EWA hydrogels. For crosslinking, wells from a 6-well plate are covered with sterile aluminum foil, CaCl_2_ solution is added and incubated at −20 °C overnight. In another 6-well plate, EWA is added and then frozen disks placed on the top to crosslink it. Next, salivary gland cells are seeded on the top of the EWA scaffolds and incubated for 7 to 10 days allowing the formation of spheroid-like structures.

Calcium chloride is one of the most common crosslinkers used in alginate gelation, which has less cytotoxicity compared to other divalent ionic crosslinkers such as Ba^2+^ [40,41,42]. These divalent cations cooperatively interact with blocks of G monomers to generate ionic bridges between different polymer chains of alginate that entraps water, eventually forming a hydrogel [43,44]. It has been shown that the gelation rate of alginate increased with temperature, while low temperatures reduce the diffusion rate of Ca^2+^ ions, leading to a slower crosslinking process, which generates a more ordered network structure, and hence enhanced mechanical properties [44,45,46]. This is crucial because for tissue engineering scaffolds, structural uniformity is important not only for well-controlled material properties but also for uniform cell distribution.

**Figure 2:**
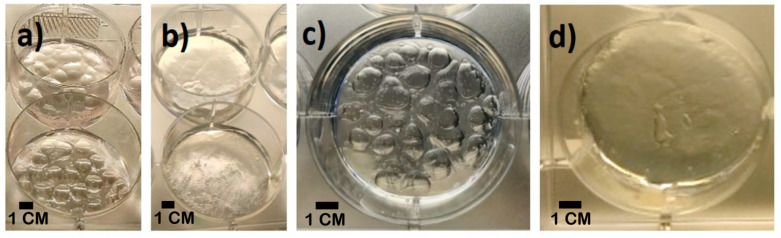
Images of EWA made by two techniques, and the small bubbles in EWA. The EWA images were acquired before (**a**,**c**) and after (**b**,**d**) the improved crosslinking technique. (**c**,**d**) show one well from the 6-well plate where the low quantity of micro and macro bubbles/pit is observed on the improved method (**d**).

At first, we added CaCl_2_ to the EWA solution by slowly pipetting the crosslinking solution directly onto the EWA. Despite dropping it carefully and slowly, pits and bubbles were inevitably produced on the surface of the EWA (Figure 2a,c). The size and depth of those pits varied and was extremely challenging to make uniform. This heterogeneity in the material macro-porosity often resulted in higher cell density at the bottom of the pits, where they clumped together while the flat surfaces had sparse amounts of cells. This could ultimately be the result of gravity, causing cells to slide down along the sidewalls of pits before they firmly attach to the scaffold.

**Figure 3:**
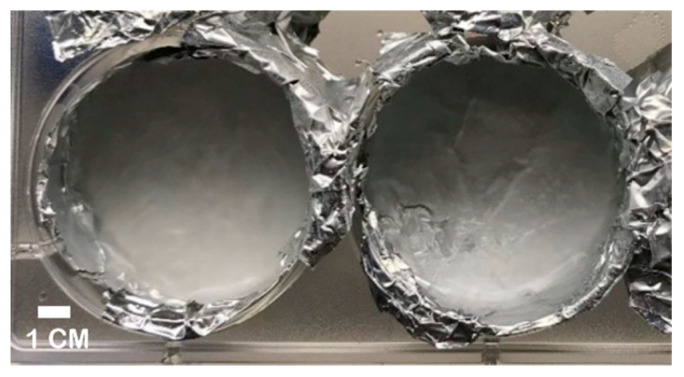
Frozen CaCl_2_ on aluminum foil pressed against the interior of the wells of a 6-well plate. Disks were pulled out vertically, ensuring the integrity of frozen CaCl_2_.

Previous work showed that the formation rate of the alginate hydrogel is higher as the working temperature rises [46]; thus, we fabricated EWA with smoother surfaces by placing frozen CaCl_2_ disks (Figure 3) on the surface of the hydrogel, allowing a slow-rate crosslinking process as the CaCl_2_ disk thawed. This method allows the formation of a smoother, uniform EWA surface (Figure 2b,d), with a visibly lower amount of bubbles within the scaffold (Figure 2d). We believe that the two main reasons leading to this improved outcome are: (a) the low temperature of crosslinking solution, which ensures the homogenous and slow-rate crosslink due to the slow release of CaCl_2_ molecules as the disk thaws; (b) the smooth surface created in the bottom surface of the frozen CaCl_2_ disks that is in direct contact with the EWA surface. We observed that after the improvement in crosslinking technique, cells and cell clumps were homogeneously distributed across the hydrogel. This improvement on EWA surface smoothness could provide more reliable results, driven by better cell distribution and cellular attachment to the scaffold.

Another challenge with using EWA is that EWA scaffolds shrink slightly during the crosslinking process due to the reorganization of the alginate chains to form a stiffer structure [13]. In our experiments, we observed an average of 15% decrease in size of the EWA scaffolds post-crosslinking, creating spaces between the hydrogel and the walls of the 6-well plate. The smaller scaffold size allows cells to be displaced off of the scaffold and instead attach to the bottom of the wells during the initial cell-seeding phase. As a result, for all cell experiments, we only included wells that contained EWA without gaps.

### 2.2. Spheroid-Like Structure Formation in EWA Hydrogel

**Figure 4:**
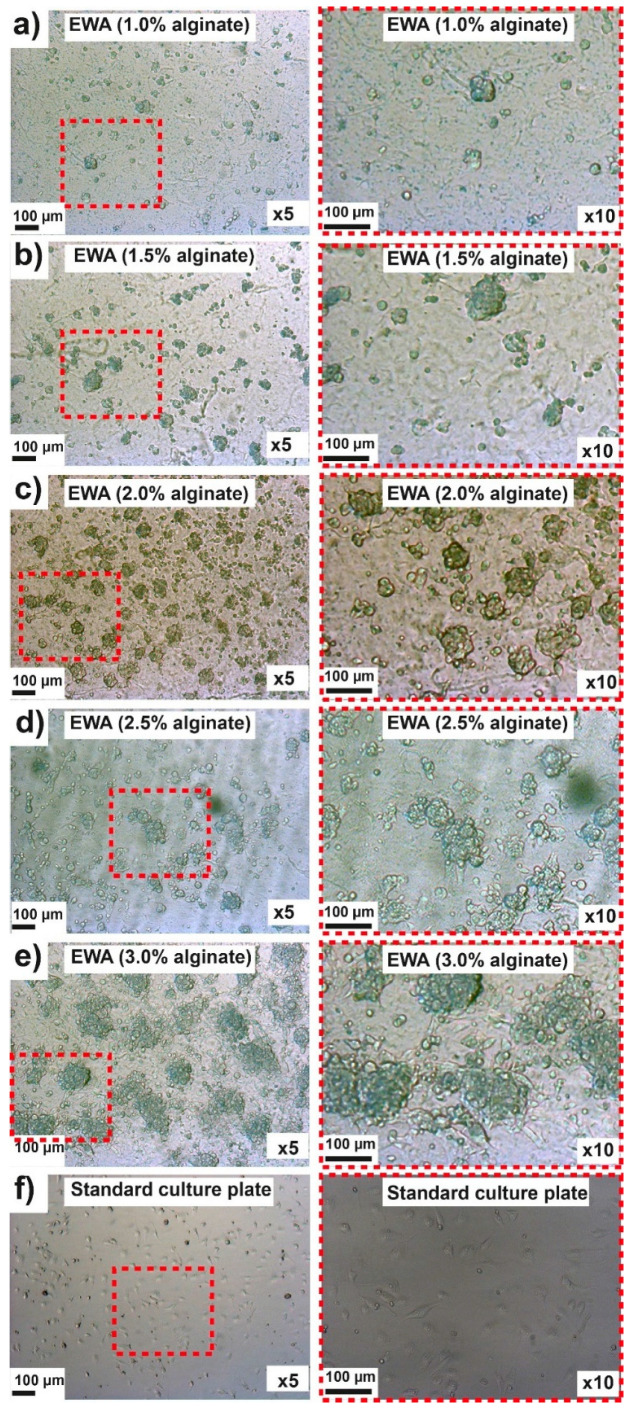
Optical images of NS-SV-AC cells growing on EWA hydrogels at day 3 after culture**.** Cells were seeded on top of the EWA at various concentrations of alginate: 1.0% (**a**), 1.5% (**b**), 2.0% (**c**), 2.5% (**d**) or 3.0% (**e**); as control, cells were seeded on a standard culture plate (**f**). Magnification ×5 (left panel) and ×10 (right panel). Scale bar 100 µm. Red dotted lines show the area of the magnified images.

Several studies have demonstrated that some mammalian cells are able to form organoid or spheroids in 3D culture, whether the cells are embedded in the hydrogel or seeded on the scaffold [47,48,49]. We evaluated the ability of five different formulations of EWA on the SG spheroid-like structure formation. Figure 4 shows the spheroid-like structure formation of the NS-SV-AC cell line seeded on EWA at day 3. Using 1% alginate, most of the cells were attached to the EWA surface and spread as a monolayer; just a few spheroid-like structures were observed on the cultures **(**Figure 4a). In the case of EWA with 1.5% alginate, more spheroid-like structures were visible (Figure 4b). 

This observable trend appears to continue with the increase of alginate percentage in the final EWA biomaterial, with 3% alginate showing the most numbers of spheroid-like structures. (Figure 4c–e). No spheroid-like structures were found in control samples (standard culture plate, Figure 4f). These results suggest that alginate concentration promotes the NS-SV-AC spheroid-like structure formation.

Similar results had been published from other research groups where cells displayed a spherical morphology in scaffolds with higher alginate concentrations, while cells in lower alginate concentrations appeared to have a spreading morphology [50,51]. Zhang et al. [50] took live-cell images on scaffolds with 0.8%, 1.3%, 1.8%, and 2.3% alginate at day 14 and found that cells in 0.8% alginate scaffolds showed a 3D interconnected cellular network, while spheroid-like structures were observed in the 1.8% and 2.3% alginate scaffolds. From our study, we observed that scaffolds with a higher concentration of alginate formed large spheroid-like structures and their sizes increased with increasing alginate concentration.

**Figure 5:**
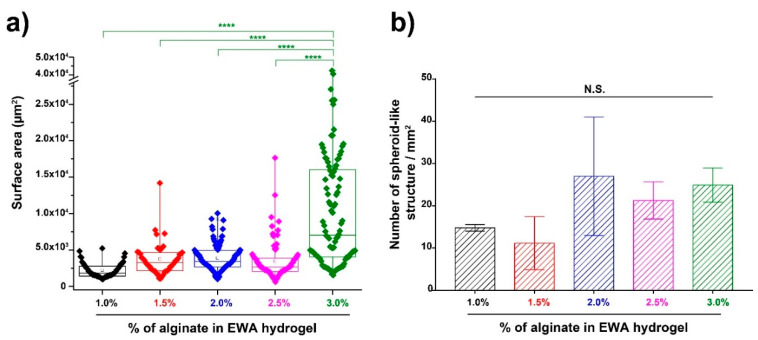
Surface area and distribution of spheroid-like structures formed on EWA hydrogels. (**a**) The surface area (µm^2^) of each spheroid-like structure found in the EWA hydrogels was measured and compared among EWA containing different alginate percentages. Data were plotted as boxplot using a box limit of 25th and 75th percentiles with a minimum-maximum whisker’s range; **** *p* < 0.0001. (**b**) The number of spheroid-like structures per square millimeter in the EWA scaffolds were determined and compared among the different alginate concentration samples. Data are presented as mean ± SD, *n* ≥ 3. N.S. not significant.

Following the characterization of the spheroid-like structure formation in EWA, we measured the surface area of the spheroid-like structures in each hydrogel. Since standard culture plates did not form any spheroid-like structure (Figure 4f), this sample was not included in the surface area analysis. The measure of the surface area is used as an additional parameter to determine the efficiency of the hydrogels in promoting spheroid-like structure formation in 3D structures [52,53,54]. Figure 5 shows the spheroid-like structure sizes and distribution in different EWA blends. We found that EWA 1% produces spheroid-like structure no larger than 5 × 10^3^ µm^2^; increasing the concentration of alginate in the EWA material resulted in larger spheroid-like structure formation (Figure 4a). We did not observe significant differences among EWA 1% to 2.5% in mean surface area; however, the EWA 3% hydrogel showed significant differences when compared with the rest of the EWA material, where spheroid-like structure sizes ranged from 5 × 10^3^ µm^2^ to 4 × 10^4^ µm^2^ (Figure 5a, green plot).

Spheroid-like structure sizes were classified based on surface area: small (500–10,000 µm^2^), medium (10,000–20,000 µm^2^), and large (>20,000 µm^2^) [52]. We found the formation of a few medium-size spheroid-like structures (between 1 and 3) in 6-well plates containing EWA 1.5%, 2.0%, and 2.5% samples, but found higher amounts of medium and large-sized spheroid-like structures in the wells containing EWA 3.0%. These results suggest that higher concentrations of alginate in EWA hydrogels promote the formation of larger spheroid-like structures.

Next, we quantified the number of spheroid-like structures per square millimeter. We did not find significant differences among the five EWA samples; however, the number of spheroid-like structures/mm^2^ in EWA 2.0% was slightly larger than the others (Figure 5b). These differences in spheroid-like structure sizes and quantities could be related to the mechanical properties provided by the alginate concentration in the scaffolds, where the stiffness of the scaffold plays an important role in cell growth, migration, and survivability [55].

**Figure 6:**
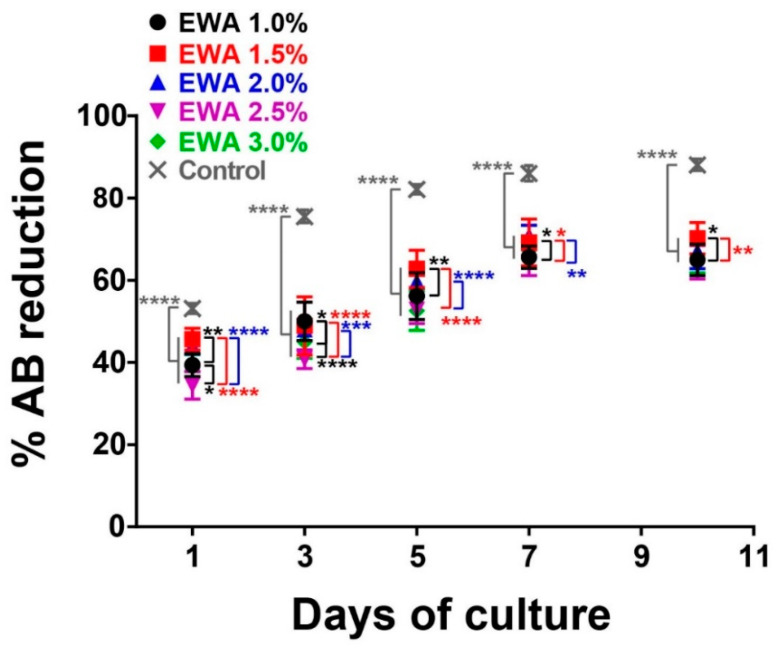
The growth rates of NS-SV-AC seeded on EWA at 1, 1.5, 2, 2.5, or 3% alginate. Cells cultured on standard culture plates were used as control. AB stands for AlamarBlue. Data are presented as mean ± SD, *n* ≥ 3, * *p* < 0.05, ** *p* < 0.01, *** *p* < 0.001, **** *p* < 0.0001.

To determine the viability of the cells and spheroid-like structures cultured in our EWA material, we performed a cell viability test using an AlamarBlue assay. AlamarBlue monitors the reducing environment of the living cell. The active ingredient is resazurin (IUPAC name: 7-hydroxy-10-oxidophenoxazin-10-ium-3-one); it is non-toxic, which allows for continuous monitoring of cells in culture over multiple timepoints. As the indicator dye accepts electrons, it can be reduced by NADPH, FADH, FMNH, NADH, and cellular cytochromes, thus changing from the oxidized, non-fluorescent, blue state to the reduced, fluorescent, pink state. Therefore, the change from oxidized to reduced state, a measure of cellular activity, can be quantitatively measured as colorimetric and/or fluorometric readings, where more detection of reduction reflects higher cell viability [56]. First, we seeded NS-SV-AC cells on EWA containing either 1%, 1.5%, 2%, 2.5%, or 3% alginate. As a control, we used cells cultured on a standard culture plate. Then we cultured the samples for 10 days, taking samples at 1, 3, 5, 7, and 10 days after the initial seeding period (day 0). Figure 6 shows the growth rates of NS-SV-AC cells seeded on EWA or standard plates. We observed that viability and proliferation increased over time in all samples from day 1 to day 7, slowing down after that, likely contributable to either the formation of large spheroid-like structures or the number of cells/spheroid-like structures nearing the maximum growth capacity of the scaffold surface area. Cells growing on standard culture plates (control samples) showed higher cell viability than EWA samples at all days of culture (Figure 6). Comparing EWA samples, EWA 1.5% showed slightly higher cell viability than the rest of the EWA, followed by EWA 1.0%, EWA 2.0, EWA 2.5%, and EWA 3.0%. This result suggests that EWA hydrogels promoted the proliferation of NS-SV-AC as well as their viability. The high cell viability on control culture is likely due to the metabolic activity that occurs in spread cells vs spheroid-like structure, where the core of the 3D structure could present lower metabolic activity.

One possible reason why EWA with 3.0% alginate showed lower cell proliferation and viability compared to the rest of the samples could also be attributed to the stiffness of the material [55]. Perhaps the stiffness of EWA containing 3% alginate is less favorable in that it does not reflect the stiffness of native SG tissue [57]; future tests should be performed comparing the stiffness of various EWA alginate percentages and the stiffness of native SG tissue. Furthermore, the formation of medium and larger spheroid-like structures in EWA 3% could also negatively impact the viability of the cell located in the core of the spheroid-like structure, resulting in a decrease of cell viability due to the self-assembly of the cell into a 3D structure [52]. Lastly, research studies have reported that cells tend to have spreading morphology and form interconnected networks when they grow on lower stiffness 3D matrix [58,59], which can drive differences in the metabolic activity of single cells and small spheroid-like structure compared with medium and large 3D structures found in the EWA 3% group [52].

## 3. Material and Methods

### 3.1. The Fabrication Procedure of EWA

#### 3.1.1. Egg White Isolation and Heat Treatment

Fresh eggs (Large White Eggs Omega-3) were purchased from a local retail store in Montreal (QC, Canada). Eggs were sprayed with 70% ethanol then were decontaminated under a biological safety cabinet (BSC). The apex of the shells was cracked and removed (an approximately 1 cm diameter hole was created). Then, the EW material was poured into a 50 mL conical centrifuge tube using forceps to pull the EW out, ensuring no visible contamination from the egg yolk; all other contents (chalaza, yolk, and watery content) were discarded. Each egg harvested provided approximately 25 mL of EW. Next, the tubes were placed in an incubator at 58 °C for 1 h to sterilize (pasteurize) the EW material.

#### 3.1.2. Egg White-Alginate Hydrogel Preparation

Sodium alginate (Protanal LF 5/60, FMC BioPolymer, Philadelphia, PA, USA, low molecular weight (398.31 g/mol)) solutions (1%, 1.5%, 2%, 2.5%, and 3%) were prepared by dissolving the alginate into 1:3 ‘Hank’s Balanced Salt Solution (HBSS, 14025076, Gibco, Burlington, ON, Canada)/Epi Max, following by shaking manually ten times. Then, the tube was placed on a Speci-Mix Aliquot Mixer (M71015, Thermolyne, Burlington, ON, Canada) in a 37 °C incubator for approximately 30 min for further dissolution. This time and procedure are enough to obtain a homogeneous solution even at 3% alginate. The samples were stored at 4 °C until use. A crosslinking solution was prepared by dissolving 90 mM calcium chloride (CaCl_2_, C77-500, Fisher Scientific, Burlington, ON, Canada) in sterile double distilled water (ddH_2_O) under a BSC.

To create the EWA, EW was poured together with sodium alginate (1%, 1.5%, 2%, 2.5%, or 3% *w*/*v*) solutions (2:1) into a 50 mL conical tube. The mixture was homogenized by pipetting. Once homogenous, the EWA mixture was centrifuged at 300*g* × 3 min at 4 °C to eliminate bubbles from the solution; the bubble foam produced on the surface was discarded. 2 mL of the EWA solution was placed into each well of a 6-well plate. Each well containing EWA was crosslinked with 90 mM CaCl_2_ solution as follows: sterile aluminum foil sheets were placed to line the bottom of each well. Next, 3 mL of CaCl_2_ solution was added into each aluminum-coated well. The plate was then placed in a freezer at −20 °C for 6 h to freeze the CaCl_2_ solution. Once frozen, the frozen CaCl_2_ disks were removed from the plate, and all aluminum foils were peeled away. Finally, the frozen CaCl_2_ disks were gently placed on the top of the EWA-coated 6-well plate, allowing the melting process to occur at 37 °C and crosslink over 12 h to create the 3D EWA scaffold. Excess CaCl_2_ solution was aspirated, and the EWA scaffolds were rinsed with PBS. The thickness of each scaffold is ≈210 mm. All procedures were performed under sterile conditions.

### 3.2. Biological Testing

The normal salivary simian virus 40-immortalized acinar cells (NS-SV-AC) [60,61] were cultured at 5% CO_2_, 37 °C in Epi Max culture medium (002-010-CL, Wisent Bio Products, ST-BRUNO, QC, Canada) supplemented with antibiotic-antimycotic (100 μg/mL penicillin, 100 μg/mL streptomycin, and 0.25 μg/mL amphotericin B) (15240062, Thermo Fisher, Burlington, ON, Canada) in a culture Petri dish (Sarstedt, St-Leonard, QC, Canada). Then, the NS-SV-AC culture was rinsed twice with sterilized PBS, and the cells were detached with 0.05% Trypsin (25200-056, Gibco) when confluency reached 90%. All biological testing with cells was performed between the 3rd–6th cell passage. 50,000 cells/well were seeded into EWA-coated plates at five different concentrations of alginate (1%, 1.5%, 2%, 2.5%, and 3% *w*/*v*), cells were also seeded on standard culture plate (without EWA) as control. Then, they were cultured with Epi Max growth medium at 37 °C, 5% CO_2_ for 10 days; the culture medium was replaced with fresh medium every 2 days. Samples were taken on days 1, 3, 5, 7, and 10, where cell viability was measured using the AlamarBlue Cell Viability Reagent (DAL1025, Invitrogen, Burlington, ON, Canada). For each well, 1.5 mL culture medium was replaced with 1.5 mL of the AlamarBlue solution. EWA-coated wells without cells were used as a control. Samples were protected from light and incubated at 37 °C and 5% CO_2_ for 4 h. We analyzed the oxidation-reduction of the AlamarBlue reagent by absorbance measurements at 562 nm and 595 nm wavelengths using 100 μL of the solution from each well and a microplate reader (EL800, Bio-Tek Instruments, Winooski, VT, USA). All experiments were performed in triplicate for every time point. NS-SV-AC spheroid-like structure formation was tracked by optical microscopy using a DM IL microscope (Leica, Concord, Ontario, Canada) at ×5 and ×10 magnifications.

### 3.3. Statistical Analysis

All test samples were performed in triplicate. Data are presented as mean ± SD. One-way ANOVA was performed with a Tukey’s post hoc test with a *P* value < 0.05. Data from the surface area were plotted as Boxplot graphs using the OriginPro 9 software (OriginLab Corporation, Northampton, MA, USA), with a box limit of 25th and 75th percentiles and a minimum-maximum whisker’s range.

## 4. Conclusions

We demonstrated that a smoother surface in our EWA hydrogel can be generated by decreasing the crosslinking rate using a frozen CaCl_2_ solution. We also demonstrated that salivary gland spheroid-like structure formation can be controlled by modifying the concentration of alginate in the EWA material. Visual differences in spheroid-like structure formation across five different EWA groups are evident, showing that the 2%, 2.5%, and 3% alginate groups of the EWA material are the better hydrogels for promoting cell self-assembly, with high cell proliferation and cell attachment. Currently, we only test the EWA hydrogel by 2.5D culture, while further improvements and tests still need to be done. For example, testing our hydrogels with a 3D extrusion printer to create geometrically defined 3D structures that could be using it for cell migration assays, estimate the porosity and polymer organization inside of the 3D structure as well as the development of spheroid-like structure inside of the EWA hydrogels (3D culture). In general, the EWA 3D culture system could be a suitable platform for future studies on drug-screening, cell migration, and as an in vitro disease model. In addition, EWA can be used as a potential source for cell transplantation (i.e., using this platform as an ex vivo environment for cell expansion). The low cost for producing EWA is an advantage.
